# Gender Difference in Academic Planning Activity among Medical Students

**DOI:** 10.1371/journal.pone.0055845

**Published:** 2013-02-13

**Authors:** Huy Van Nguyen, Thao Thach Giang

**Affiliations:** 1 Department of Health Organization and Management, Institute for Preventive Medicine and Public Health, Hanoi Medical University, Dong Da Dist, Hanoi, Vietnam; 2 Vietnam National Hospital of Pediatrics, Dong Da, Hanoi, Vietnam; Northwestern University, United States of America

## Abstract

**Background:**

In Vietnam, as doctor of medicine is socially considered a special career, both men and women who are enrolled in medical universities often study topics of medicine seriously. However, as culturally expected, women often perform better than men. Because of this, teaching leadership and management skill (LMS) to develop academic planning activity (APA) for female medical students would also be expected to be more effective than male counterparts. This research aimed to compare by gender the effect of teaching LMS on increasing APA, using propensity score matching (PSM).

**Methods:**

In a cross-sectional survey utilizing a self-reported structured questionnaire on a systematic random sample of 421 male and female medical students in Hanoi Medical University, this study adopted first regression techniques to construct a fit model, then PSM to create a matched control group in order to allow for evaluating the effect of LMS education.

**Results:**

There were several interesting gender differences. First, while for females LMS education had both direct and indirect effects on APA, it had only direct effect on males’ APA. Second, after PSM to adjust for the possible confounders to balance statistically two groups – with and without LMS education, there is statistically a significant difference in APA between male and female students, making a net difference of 11% (p<.01), equivalent to 173 students. The difference in APA between exposed and matched control group in males and females was 9% and 20%, respectively. These estimates of 9.0 and 20.0 percentage point increase can be translated into the practice of APA by 142 males and 315 females, respectively, in the population. These numbers of APA among male and female students can be explained by LMS education.

**Conclusions:**

Gender appears to be a factor explaining in part academic planning activity.

## Introduction

Gender difference in academic performance has become difficult to overlook. Most studies show that, generally, girls do better in school than boys. To be specific, girls achieved higher grades and completed high school at a higher rate compared to boys [1], [2], [3]. Standardized achievement tests also show that females were better at language and literature [4]. Recently, an international aptitude test administered to fourth graders in 35 countries showed that females outscored males on reading literacy in every country [1]. Although girls often exhibit higher verbal ability throughout high school, they begin to lose ground to boys after fourth grade on tests of both mathematical and science ability [1].

What is about such a trend in higher education settings? According to Dayioglu and Türüt-Asık, when female students managed to enter the university, they entered with lower scores [5]. However, once they have been admitted to the university, they excelled in their studies and outperformed their male counterparts [5]. If so, what are the factors that give rise to these differences? Prior literature has identified that academic performance is affected by a variety of factors. These include individual and familial characteristics such as student ability, motivation, the quality of education obtained and the like [5]. The gender of the student may also be one of the determinants. Childhood training and experience, gender differences in attitudes, parental and teacher expectations and behaviors, differential course taking and biological differences between the sexes may all be instrumental in explaining gender differences in achievement [5].

In terms of course and topic choices, in most countries, there is also a gender difference in the type of courses students select and in academic performance. As in a study by Eccles [6], females are less likely than males to enroll in courses in the physical, computer, and engineering sciences. Males are less likely than females to enroll in courses in foreign languages, literature, art, and the humanities. These gender differences in academic choices begin to emerge in secondary school and continue during college and university - leading to a gender difference in college majors and in occupational choices. In Vietnam, the same is also true where male students prefer natural sciences such as math, physic, chemistry, medicine, etc while female counterparts like to study social sciences – literature, history, geography, language, etc. Across the time, there is one interesting change. In the past, most students enrolled in schools of medicine were males, but now there is even more female students’ face. The question of concern here is not how many male and female students would there be, but who performs better? In Vietnamese medical schools, students are required to study and pass all three groups of topics, basic sciences, public health, and clinical sciences. Among the public health topics, health organization and management including leadership, management and planning skills (LMS) for healthcare activities are taught to students. Based on our observation over the past 20 teaching years, male students appeared to prefer studying LMS than female counterparts as they thought it could prepare them necessary leadership and management skills to become a leader or a manager of their healthcare facilities. A question emerged here is whether female students better perform this topic even though they seemingly do not like this topic than male counterparts.

In Vietnam, most studies examined students’ academic scores as main indicators to measure the effectiveness of teaching, while intermediate behaviors like academic planning activity (APA) of students which help them achieve higher academic progress, have remained in question. APA is increasingly important as students have been claimed under pressure in terms of time management due to a variety of theoretical and practical topics enrolled simultaneously in the same year. It is further pressure placed on students in terms of academic achievement due to a great volume of knowledge and skills to be learned within universities. To assess the impact of an education program, researchers and evaluators may adopt different methods, one of which is PSM, the powerful statistic [7], [8], [9], [10], [11]. This method can help to reduce selection bias because it allows for quasi-experimental contrasts between students in receiving “treatment” and “control” groups based on their observed characteristics. Because of its ability to reduce selection bias, PSM is commonly used in the fields of education and communication [12], [13].

**Figure 1:**
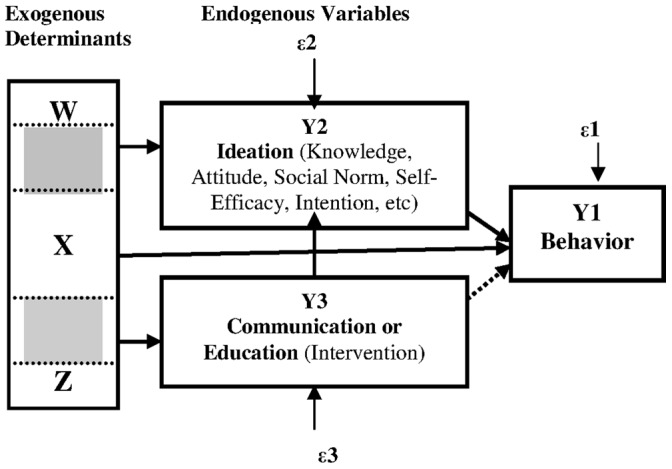
A Model of Behavior Change Communication.

A conceptual model for this research relies upon a model comprehensively adapted by Kincaid [8], [9] (Figure 1). In this theory, the psychological influences including knowledge, attitude, social norm, intention, self-efficacy, and others can be combined as ideation. Specific education interventions may be designed to influence only one or several types of psychological processes. All sorts of psychological processes are expected to influence behavior even if communication is designed to influence only one of them. Education or communication affects behavior indirectly by providing information that changes one or all of such processes. Exogenous determinants including demographic, socioeconomic and contextual characteristics affect endogenous variables – such as the exposure to the communication, ideation and behavior.

Because in many academic settings teaching a particular topic is applied to every student enrolled in the same academic year, it is a difficult task for researchers to design a randomized control group study. With the use of PSM technique, it is possible to create a matched control group so that we can justify the influence of an education program [14].

The purpose of this research was to compare by gender the effect of teaching LMS on increasing APA, using propensity score matching (PSM).

## Methods

### Study Design and Setting

A cross-sectional sample survey was conducted using a self-administered structured questionnaire. The study was carried out in Hanoi Medical University (HMU), located in the Northern country, one of the leading universities and a leading medical education institution among eight medical universities in Vietnam. It is well situated to undertake this proposed assessment. For many years, HMU has been a focal point for networking and disseminating innovations in medical education and medical research. Similar to all eight medical universities, HMU offers teaching LMS topic with the same contents designed to help students develop leadership and planning skills, which are helpful to their current study and their future health care career.

### Participants and Sampling Procedure

A total of 421 students across academic years 1–6 were surveyed from November to December 2009 using a representative systematic random sample from a sampling frame of 3145 students, a total population size of undergraduate students from the first to sixth year. The sampling frame was provided by the Head of the HMU Undergraduate Training Department. Following a random first number, 421 students were selected, using a sampling interval of 7.

### Data Collection

The instrument for data collection was a self-administered structured questionnaire, comprising four sections, first asked students about their individual and social characteristics, second the level of access to LMS education, third knowledge, attitudes and self-efficacy of LMS and finally APA during the past and current year.

As a procedure, once the study was approved by the HMU Undergraduate Training Department, all selected students were informed of the study objectives and contents. 95% of the sample gave their informed consent to participate; because 5% refused the study, we used the same sampling strategy to approach more students until a full size of 421 students was reached. Both male and female students were surveyed, using the anonymous self-reported manner. Each student completed a confidential questionnaire for 15 to 20 minutes either before or after a lecture he or she attended as well as after permissions received from teachers.

This study was informed by a pilot survey to validate the instrument. The pilot showed that the instrument was technically feasible for the main survey.

### Measurement

Exposure to LMS education was measured as a state whether a student has learnt LMS topic. For more reliable and valid measure, the recall of key contents at the interval level of measurement was employed. To accommodate this measurement, an open-ended question was added to ask students to remember and list all possible contents of lessons they have learned about LMS. To transfer textual data into standard contents, a principal investigator who knew well about the LMS topic and its contents, closely looked at all textual responses, then classified if each of them would go under the standard contents of the topic. In total 8 standard contents were identified. The above question formed a continuous scale measuring the level of recall that ranged from 0 to 8 contents with a median of 4. To simplify this measurement and ease the logistic regression and propensity score analysis, the scale was classified into 1 and 0 (higher versus lower recall).

LMS knowledge was assessed by 8 true/false/don’t know items such as “*a good academic planning includes setting goals and considers timeline frame and resources*”. Scoring the knowledge scale was accomplished by dichotomizing each item into a value of 1 (correct) and 0 (incorrect or don’t know) and then summing the item values to form a composite score with higher scores reflecting better knowledge (Cronbach’s alpha = .50; mean score = 6.32, SD = 1.96). Attitude toward LMS was measured with seven 5-point semantic scale (*bad–good)* from 1 (negative evaluation) to 5 (positive evaluation) such as “*how good or bad would it be if you talked about LMS for each topic with your friends every year?*” A composite score was obtained by summing responses to items with higher composite scores indicating higher levels of attitudes (Cronbach’s alpha = .55; mean score = 16.26, SD = 3.76). Social norm toward LMS was assessed with seven 5-point semantic scale (*untrue-true)* from 1 (negative evaluation) to 5 (positive evaluation) such as “*Most people - family, teachers and close friends - who are important to you think you should talk about APA with your friends every year*?” A composite score was obtained by summing responses to items with higher composite scores indicating higher level of social norm (Cronbach’s alpha = .85; mean score = 13.57, SD = 4.30). Intentions for APA are measured by asking students to rate on a 5-point semantic scale ranging from *very unlikely* (1) to *very likely* (5) such as “*during the next few months, you intend to talk about academic planning with your friends for each topic?*”. A composite score was formed by summing responses to items with higher scores indicating higher levels of intentions (Cronbach’s alpha = .83; mean score = 15.62, SD = 5.17). Self-efficacy of academic planning was measured with seven items tapping perceived difficulty of academic planning on a 5-point semantic scale from *very hard* (1) to *very easy* (5) such as “*how hard would it be for you to advice or persuade your friends to make academic planning?*” A composite score was obtained by summing responses to items with higher scores reflecting higher levels of self-efficacy (Cronbach’s alpha = .63; mean score = 18.61, SD = 5.10). These five related sub-constructs representing the cognitive and social interaction component of ideation were used to construct the measures of ideation. For the logistic regression analysis, using a cut-off of 50% the measures were split into 1 and 0, corresponding to higher and lower levels of ideation.

One of the outputs of LMS education was the past year and current APA which were combined to construct a continuum of behavior with the following five scale values: *0 = never, 1 = rarely, 2 = occasionally, 3 = usually,* and *4 = always* (Cronbach’s alpha = 0.70). Combining these two items into a single outcome variable has two advantages in that it makes the measurement more valid and reliable as well as allows the analysis of the impact of education on students’ APA. The levels of APA met the minimal requirements of order with respect to ideation with the support from one-sided test of significance performed with *p* = .01 [15]. This level of probability indicated the rejection of the null hypothesis of equality of levels and supporting the alternative hypothesis of order. To make logistic regression analysis and estimation of the impact possible, the scale of the single APA was classified as 1 and 0 reflecting more and less frequent level of APA.

Socioeconomic status (SES) was measured by means of living total money received from family categorized into two levels – higher and lower socioeconomic status. Age was measured by number of years categorized into higher and lower groups. Level of university education was measured by ordinal number of academic years categorized into seniority (three final academic years) and juniority (three first academic years). Origin of permanent residence was classified into urban and rural area. Demographic characteristics including gender, ethnics and religion were also included. Further, the literature has indicated that a student’s father and/or mother occupation – called parental occupation (white vs. blue collar) predicts his or her study behaviors and academic achievement [16], [17], [18]. To maximally reduce the potential for selection bias, we included these covariates in the model predicting propensity to receive education [19].

### Statistical Analysis

The software program “STATA version 10.0” was applied to all the statistical procedures.

#### Simple proportion differences

Chi-square tests were used to determine whether proportion differences in APA between female and male students and between before and after adjustment with PMS in each group were statistically significant. We used a *P* value of.05 for these analyses. We considered the results obtained from Chi-square tests of these proportion differences as a “benchmark” for the results obtained from analyses using PSM as mentioned below.

#### Logistic regression modeling

The model of direct and indirect effects used for this analysis requires three regression equations, one for each endogenous variable: APA, ideation, and recall of LMS contents educated. Each of such equations may have an error or a disturbance, e1, e2 and e3 for APA, ideation and education, respectively. Because APA, ideation, and exposure are measured with a binary scale, logistic regression is used to estimate parameters of the equation. The differentiation among the X, Z, and W matrix of exogenous control variables indicates that each endogenous variable should be determined by exogenous variables not included in the other two equations. However, some overlap of exogenous variables can be acceptable [20]. The arrows indicate the hypothesized direction of influence and effects, that the three error terms are uncorrelated, and that there is no third unobserved variable that accounts for any of the hypothesized relationships. The model fit of these three equations can be secured if there are not any unobserved variables responsible for the observed relationship between exposure and the outcome, meaning that if the disturbance terms, ε1, ε2 and ε3 are statistically uncorrelated as reflected as rho with *P*>0.05 or CI95% does not contain a value of 1.0. And if so, y3 can be regarded as exogenous, and simple regression can be adopted [11].

#### Calculating actual difference between education and matched control using PSM

A propensity score is the probability of being exposed to an education or an intervention given a set of observed covariates, *W, X and Z*. The method as this was developed as a means to balance the education and control units so that a direct comparison would make a valid conclusion. For research survey, a single score for matching is generated using statistically regressing exposure on all of the variables that determine exposure and also may be related to the outcome variable [11].

As a process of data analysis, after running the logistic or probit regression model to calculate all respondents’ propensity for experiencing an education program of interest, in this case, receiving LMS education, we then used the estimated propensity scores to match students who did and did not receive LMS education. Finally, the STATA 10.0 respective command was adopted to calculate actual difference in APA between education group and matched control group after adjusting for possible confounders.

#### Research ethics

The Institutional Review Board (IRB) of the HMU did not require a formal ethical approval for the survey as this study was a cross-sectional survey on a non-sensitive topic, and students were fully voluntary to participate.

## Results

### Sample Characteristics

Among 421 undergraduate students involved in the study, each group of male and female students accounted for around 50%, including academic years 1 through 6, each contributing up to 16.5% of the total. The proportions were fairly evenly distributed by the origin of permanent residence (urban vs. rural, each ∼ 50%). The mean age of all participants was 21 years (range 18–27 years: SD = 1.89). The mean amount of monthly stipend from family for university life and study was VND1.36 million (range = .3–10: SD = .72; US$1 = VND19,000). The main differences were in ethnicity (most were the Kinh, a major ethnic group in Vietnam), religion (most were the followers of Buddhism, ancestor worship and non-religious), and parental occupation, with white collars making up almost 70% of the total.

### Simple Proportion Differences

In Table 1, data compare APA proportions between the study’s subsamples of those exposed to and not exposed to LMS education and between female and male students. As seen, there was no difference in APA between female and male students either when exposed or not exposed to LMS education. However, there was statistically a significant difference in composite APA between exposed and non-exposed groups among female students (*P*<0.001) as well as among male counterparts (*P*<0.01). Overall, female students who had a higher recall of LMS contents showed a higher proportion of APA compared to those with a lower recall (74.67% vs. 42.00%; with a relative gain of 32.67 percentage points). The same was also true for male students with a difference of 24.10% (66.67% vs. 42.57%). Also, there was a significant difference between female and male students in terms of the relative gains in APA (32.67% vs. 24.10% in favor of female; *P*<0.01).

**Table 1:** Dependent variable proportions for LME students and non-LME students.

Dependent Variable (APA)	Exposed Students n(%)			Non-Exposed Students n(%)			*P* (χ2)	
	Enrolled in LME			Non-Enrolled in LME			§ (Female)	(Male)
	Female (n = 86)	Male (n = 60)	*P* (χ2)	Female (n = 139)	Male (n = 136)	*P* (χ2)		
APA during the past year	38(44.19)	27(45.00)	NS	42(30.22)	43(31.62)	NS	*	NS
APA during the current year	39(45.35)	30(50.00)	NS	49(35.25)	50(36.76)	NS	NS	NS
	**Exposed to 50% or more of all LME sessions**			**Exposed to just less than 50% of all LME sessions**				
	**Female (n = 75)**	**Male (n = 48)**	***P*** ** (χ2)**	**Female (n = 150)**	**Male (n = 148)**	***P*** ** (χ2)**		
APA during the past year	33(44.00)	24(50.00)	NS	47(31.33)	46(31.08)	NS	NS	*
APA during the current year	35(46.67)	26(54.17)	NS	53(35.33)	54(36.49)	NS	NS	*
	**Higher recall**			**Lower recall**				
	**Female (n = 75)**	**Male (n = 48)**	***P*** ** (χ2)**	**Female (n = 150)**	**Male (n = 148)**	***P*** ** (χ2)**		
Composite APA	56(74.67)	32(66.67)	NS	63(42.00)	63(42.57)	NS	***	**

§ comparison between females exposed and non-exposed;

¶ comparison between males exposed and non-exposed;

* *p<.05;*

** *p<.01:*

*** *p<.001;*

*NS = not significant.*

### Logistic Regression Modeling for Predictors of Recall, Ideation and APA for Female and Male Students Separately

Table 2 shows the results of the logistic regression analysis using probit procedure. Descriptive statistics for each variable for female and male students are reported in the second and third column. The equations for recall, ideation, and APA were identified with 8 exogenous variables. Results of the exclusion tests are reported at the bottom of the column for each equation. No statistically significant effects of exclusion were found. The proportion of the variance explained by the equation for recall, ideation, and APA was 70, 12, and.13, respectively for female students and.29, 11 and.10, respectively for male counterparts. For female students, recall of LMS contents had a direct, significant effect on APA (.85, *P*<.001) as well as an indirect effect through its effect on ideation (probit coefficient = 1.06, *P*<.001). However, it is not the case for male group, where recall of LMS had only a direct effect on APA (probit coefficient = .69, *P*<.01).

**Table 2:** Results of the structural equation modeling for APA by gender.

Variables	Description % or mean (range)		Model 1 Logistic regression (probit procedure)					
			Recall (Probit coef.)		Ideation (Probit coef.)		APA (Probit coef.)	
	Female	Male	Female	Male	Female	Male	Female	Male
Age (older vs. younger)	20.99 (18–26)	21.1 (18–27)	1.60***	0.75***	−0.16*	−0.36**	–	–
Parental occupation (white vs. blue collar)	42.22	39.80	−0.13	0.31*	0.29	0.49*	–	–
Place of permanent residence(urban vs. rural)	48.89	45.41	0.55*	−0.10	–	−0.29	–	–
Ideation (higher vs. lower)	16.89	19.39					1.06***	0.43
Recall (higher vs. lower)	33.33	24.49			0.83**	−0.21	0.85***	0.69**
Academic year (senior vs. junior)	54.22	48.47	–	–	–	0.69*	–	–
Living stipend level (millionVietnamese Dong)	1.35 (0.4–10)	1.37 (0.3–4)	0.45*	0.15	0.18	−0.13	–	–
Religion (yes vs. no)	2.67	1.53	–	–	1.19*	–	–	–
Number of cases	225	196	225	196	225	196	225	196
Variance explained (adjusted R^2^)			0.70	0.29	0.12	0.11	0.13	0.10
Exclusion test (model fitness) (*p*)^1^			NS	NS	NS	NS	NS	NS

Test for endogeneity - Biprobit rho for Model 2: −0.12 (−0.83–0.74) for females and −0.19(−0.02–1.03) for males.

rho-based *p:* Chi^2^ = 0.051; p = 0.821 for females and Chi^2^ = 2.20; p = 0.13 for males.

Statistical significance: *p<.05; **p<.01; ***p<.001; NS = not significant.

Exclusion of a variable for model identification is indicated by (−).

1 The likelihood ratio test was used for binary dependent variables.

### Model Fit

The model fit was examined with two tests. The first was to test for endogeneity and support for the assumption of strong ignorability of endogeneity of exogenous variables in endogenous variables. To do this, we treated the problem as a two-equation system. The results showed that high recall of LMS contents had a statistically significant direct effect on APA (probit coefficient = .85; *P*<.001 for female and.69; *P*<.01 for male) after controlling for the remaining variables in the model. Biprobit analysis of these two equations was conducted to test for the endogeneity of level of recall in the APA equation. The correlation of the error terms was not statistically significant (rho = −.12; *P*>.05 for females and = −.19; *P*>.05 for males), indicating that LMS recall may be considered as exogenous in the equation of APA. It also means that when the error term from the equation for recall was added to this equation, it was not statistically significant as well. This suggests that ordinary probit regression can be used to estimate the direct effects of high recall of LMS contents and that the potential effects of omitted or unobserved variables may be ignored. The second was to test for exclusion of a variable as shown at the bottom line of Table 2. We can argue that the model fits well the data as *P* value of all of the models for female and male groups was not significant. The result of the propensity score analysis as informed from these equations is therefore expected to approximate what we would expect from a randomized control group design.

Table 3 shows the results of the stratification of propensity score. We used stratum-based PSM in order to maintain the full sample design and yield results based on the same cases as the structural equation modeling (SEM) analysis. The propensity score – probability of high recall - ranged from.002 to 1.000 with a mean of.62 (SD = .36) for female students and from.03 to 0.98 with a mean of.48 (SD = .20) for male counterparts. The continuous score was stratified into 5 and 7 balanced subgroups for female and male students, respectively, within which there were no statistically significant differences in the propensity between those with high recall (treatment group) and those with no/low recall (matched control group). For female students, results of the propensity score analysis identified that 74.67% of participants in the treatment group (with high recall) engaged in APA after the LMS education compared to 54.67% of participants in the matched control group (with low/no recall), a statistically significant difference of 20.00 percentage points (Z test-based *P*<.05). For male counterparts, the corresponding proportions were 66.67% and 57.67%, making a significant difference of 9.00 percentage points (Z test-based *P*<.05). Notably, after PSM-based adjustment, there was statistically a significant difference of 11% in net difference in APA between female and male students (20% vs. 9%; *P*<.01).

**Table 3:** Balance of the Propensity Scores for LME exposure: Results from PSM.

Groups	Non-Exposed to LME (A) N	Exposed to LME (B) N	Mean Propensity Score±SD (Range)	Number of Balanced Blocks#	Net Difference (B-A) Using “Atts” Command
Female students (N = 225)	150	75	0.62±0.36 (0.002–1.000)	5 blocks	20.0% (*P*<0.05)
Male students (N = 196)	148	48	0.48±0.20 (0.03–0.98)	7 blocks	9%(*P*<0.05)
*P* (χ2) for comparing proportions between different female and male groups					*P*<0.01

# pscore statistics indicate that there is no statistical difference between A and B within each stratum (p>.05), meaning that propensity scores balanced at 5 strata for females and 7 strata for males.

**Figure 2:**
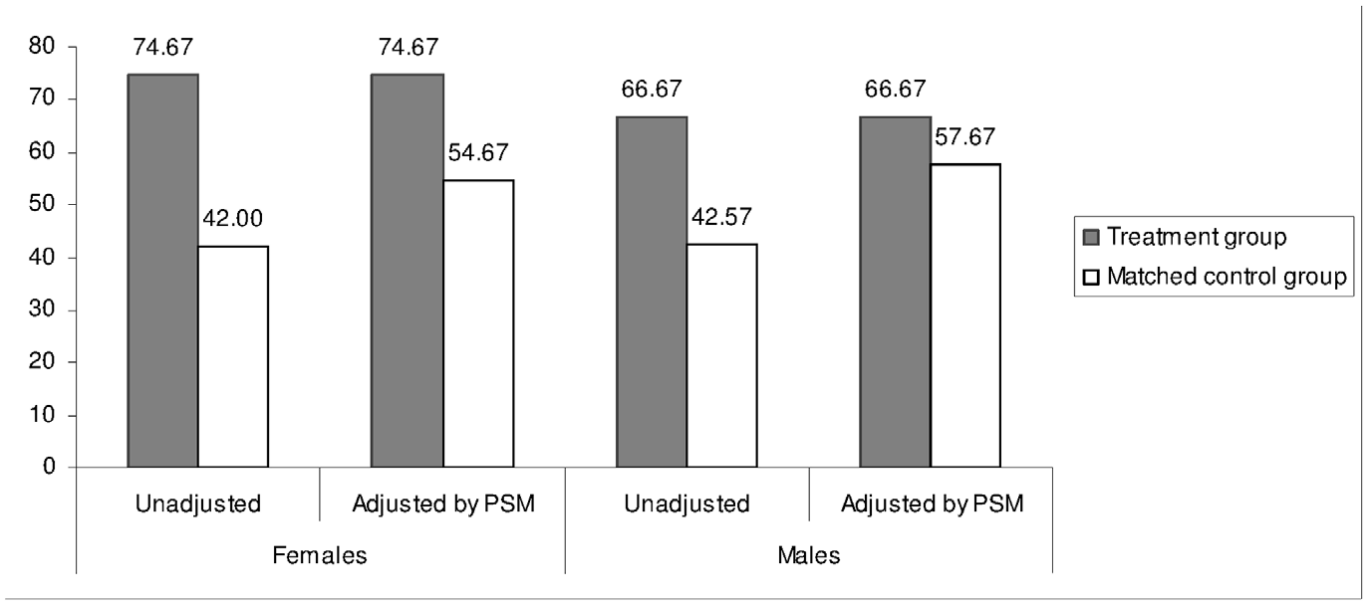
Comparison of the unadjusted increase in APA to the increase adjusted by PSM.

In Figure 2, the unadjusted difference in APA between the treatment group and matched control group was 32.67 percentage points (74.67% minus 42.00%) for females and 24.10 percentage points (66.67% minus 42.57%) for males. The propensity score results were obtained as a result of the weighted sum of differences in APA among the balanced, statistically equivalent subgroups. The adjusted difference between the treatment group and matched control group was 20.0 percentage points (74.67% minus 54.67%) for females and 9.00 percentage points (66.67% minus 57.67%) for males.

## Discussion

One of the major missions of education is to help students develop knowledge and skills appropriate to and necessary for their future careers. However, academic activities during the school life have usually been better performed among female rather than male students in most topics in many countries [2], [3], [21]. We question here if it is true for the topic, LMS education, which has been offered at HMU, a leading university among eight medical schools in Vietnam. This topic is considered important as it can help medical students to develop leadership and management skills such as APA and organizations’ planning skills which are necessary for their healthcare careers. This research aimed to compare APA as well as the effect of teaching LMS on increasing APA between female and male students. Because we could not randomly assign students to receive or not receive LME, we used propensity score matching (PSM) techniques to contrast the behavior of students who did and who did not receive this education but who had been matched on a variety of observed background characteristics. The study revealed a number of interesting differences by gender.

### Gender Simple Difference in APA and Model Predicting APA

We found that although men and women did not differ in APA either when they were exposed or not exposed to LMS education, in both female and male groups, exposed and non-exposed students differed in composite APA. Medical students - regardless of being females or males - who had a higher recall of LMS contents showed a higher proportion of composite APA compared to those with a lower recall. Notably, there was a significant difference between female and male students in terms of the relative gains in APA (32.67% vs. 24.10%). This suggests that female students likely demonstrate a better performance of studying LMS than male counterparts. Unfortunately, data on students’ APA behavior from the present study are not comparable internationally as well as in Vietnam because there is no previous research examining this matter among university students. However, this difference in the relative gain of unadjusted APA between female and male students in favor of female counterparts can be anticipated as it is quite consistent with other studies on many topics other than LMS or APA among junior and senior school students [1], [2], [3], [5], [6], [18], [21], [22]. Interpretations of this difference between females and males could be problematic as Morgan claimed [23] if we use only the simple statistical statistics (univariate analysis) such as Chi square test for contrasting this effect.

In terms of the multivariate model predicting APA, there are also several important differences between female and male students. In female group, it was found that both LMS recall and ideation was related to APA, while for male counterparts only one factor, LMS recall, was. Female students who had higher recall of LMS messages and had higher perception or ideation of LMS were more likely to engage in APA. To male counterparts, those students who recalled more contents of LMS were more likely to exercise APA. As such, the after-only, cross-sectional regression analysis without ideation showed a significant direct effect of the education campaign on the adoption of a behavior after controlling for socioeconomic variables. This is as much as many studies of mass media impacts are able to do [7]–[11]. However, for female students when ideation was added to the after-only regression equation of APA, the direct effect of education program remained statistically significant. This result confirms the indirect effects of the education, but also its direct effect on behavior, emphasizes the role of mediating effect of ideation, and provides support for the theoretical model that guides the design of education or intervention and evaluation of the results as suggested by Kincaid’s studies in Philippines [7], [8], [9], [11]. This means that teaching LMS has changed LMS perception (or ideation) of the female students first, and then influenced their behavior.

On the contrary, we found the evidence of the only direct effect of education program on APA among male students. As can be seen in Table 2, the effect of LMS recall on ideation was not significant, but on APA was, and the effect of ideation on APA was not significant. This suggests that teaching LMS for male students appears working directly on APA. Understanding this difference between female and male students in terms of the impact of education program can help design an appropriate intervention program which is specific to female and male group, separately.

### The Role of LMS Education and Gender Difference in the Actual APA

After controlling for background variables, the LMS recall had a significant effect on both ideation and APA, and ideation had a significant effect on APA among female students; the LMS recall had a significant direct effect on APA among male students. The potential effect of unobserved variables (not in the equations) and the reciprocal effect of behavior on ideation and recall can be ruled out given the results of the statistical tests for endogeneity. The only criterion missing for a causal inference was a counterfactual condition which could have been provided only in a controlled experimental design. In our study, the counterfactual condition was made by PSM in order to create a matched controlled group so the comparison of net difference could be made possible. However, acceptance of such a causal inference for teaching LMS and ideation on APA does not necessarily mean that other causes were not also acting. Students could approach or may be exposed to other sources of LMS education such as internet, library or others. But at least in this study it is quite possible to hold that the effect on APA can be explained by LMS education per se which was designed and offered by the Department of Health Organization and Management at HMU. This is because we measured the recall of the key contents of LMS taught only at the university.

Looking at the effect of teaching LMS on APA for female students, comparing between treatment group and matched control group, a 20.00 percentage point increase in APA after the education delivered may sound small. However, because the female sample of 225 can represent a total population of 1572 female students (this number was provided by the Department of Undergraduate Training from HMU), the actual net increase in the number of female students practicing APA is estimated to be 315. The propensity score results for male counterparts detected a 9.00 percentage points that can be translated into the practice of APA of 142 male students. As data about APA among students before are not available, we are able to compare the net change of APA with that of other behaviors among other populations. It was found that the proportions of the actual APA change among our sample are in mid-range as compared with other studies [7], [11]. A special note worthy to concern is that there was a significant difference in the net gain in APA between female and male students (20% vs. 9%), making a net difference of 11%. This can be translated into a difference in APA of 173 female students as compared with male counterparts that can be attributed to the impact of LMS education.

### Why a Gender Difference in APA

In Vietnam although changed over time, social norms that favour men in higher social class remain existent. In the past, rooted in the family, women were often expected to be centered home to care for their family, while men mainly performed productive tasks – higher positioning and generating income. Women were not encouraged or even not trained to become a leader or a manager of a society, while men were socially accepted to do so. The role of women in the society was limited. To date, the situation on gender relation appears in a process of change, although according to Phinney [24], most Vietnamese men remain concerned with choosing for marriage a wife who can provide them with an economically stable, happy, and harmonious home conducive to raising children rather than being engaged in the society. On a positive note, however, women now have more opportunities and choices to join social activities and social positions. In medical schools before, more men than women took up leadership and management as a topic for study because they wanted to be prepared for the future post of leader or manager, but now women are increasingly enrolled in this topic. Women are encouraged to be more in the higher position. As they have this kind of interest, it may help them better perform than men.

The second explanation would be based on gender theory. According to this theory, males and females enter the educational system with different sets of behaviors, attitudes and values [25]. These gendered behaviors, attitudes and values are the result of childhood socialization in line with the cultural norms of masculinity and femininity [21]. It is proposed that, in educational settings, male behavior, values and attitudes interfere with males’ educational achievement. For example, Warrington et al. found that boys were more likely than girls to be ridiculed by their peers for working hard at school, and frequently resorted to ‘laddish’ behavior such as challenging authority, drawing attention to themselves and pretending not to care about schoolwork in order to gain acceptance from their peer group [26]. These phenomena remain pervasive in the Vietnamese context.

Another factor could be that schools adopt learning and assessment procedures that appear better suited to females rather than to males [25]. These arguments assume that males and females possess different sets of behaviors, attitudes and learning styles and thus require different school and teaching practices to succeed. In some cases, these explanations claim that teaching and schooling has become ‘feminised’ and some schools are no longer adequately addressing boys’ educational needs [25].

### Limitations

The present study could have several limitations. Due to the nature of self-reported design, recall bias would be inevitable. Some students, perhaps because of their self-esteem, could over- or under-estimate their behavioral responses. However, as this study was designed with a survey on a fairly large, representative sample with anonymous and confidential commitment, it would partly reduce that bias. Also, as a cross-sectional study, it may limit our ability to infer the order of causality; therefore, a longitudinal study is needed to address this concern. For example, a number of repeated surveys among students or a follow-up study on the same students over time could be helpful to determine the temporal relationship between LMS education and APA.

### Conclusions and Implications

Despite several limitations, this study is the first to examine if there is gender difference in APA and if the effect of teaching LMS on APA is greater among female or male students. It was found that men and women differed in several important dimensions. Generally, female students better performed in APA than male counterparts both before and after adjustment with PSM. LMS education had direct effect on APA, but also had indirect effect on APA of female students through ideation. For male counterparts, LMS education had only direct impact on APA. These differences can be explained by gender theory, school factors and a changing society with social norms that appear to favor females. These results suggest that designs that aim to improve male academic performance should include education programs that can directly influence an academic outcome or behavior. However, care should be given when tailoring up an education program or plan to increase academic activity for females. It could be helpful to create education programs or contents that can change perceptions or ideation for women before we wish to change an academic behavior such as APA. Additional efforts to change the situation could consider ways to challenge gender factors and social norms in a manner that both men and women should be equally supported. To do this, maybe one more step should be taken. It is recommended that researchers or programmers conduct a qualitative study to identify what and how they change the current situation. If the model is successful, it can be rolled out to other medical schools in Vietnam or to similar contexts of developing countries.
